# Emerging and Established Trends to Support Secure Health Information Exchange

**DOI:** 10.3389/fdgth.2021.636082

**Published:** 2021-04-09

**Authors:** Emmanouil G. Spanakis, Stelios Sfakianakis, Silvia Bonomi, Claudio Ciccotelli, Sabina Magalini, Vangelis Sakkalis

**Affiliations:** ^1^Computational Biomedicine Laboratory, Institute of Computer Science, Foundation for Research and Technology–Hellas, Heraklion, Greece; ^2^Department of Computer, Control, and Management Engineering Antonio Ruberti, Università degli Studi di Roma La Sapienza, Rome, Italy; ^3^Emergency and Trauma Surgery Unit, Fondazione Policlinico Universitario Agostino Gemelli, Rome, Italy

**Keywords:** interoperability, health information exchange, eHealth, blockchain, security, patient consent

## Abstract

This work aims to provide information, guidelines, established practices and standards, and an extensive evaluation on new and promising technologies for the implementation of a secure information sharing platform for health-related data. We focus strictly on the technical aspects and specifically on the sharing of health information, studying innovative techniques for secure information sharing within the health-care domain, and we describe our solution and evaluate the use of blockchain methodologically for integrating within our implementation. To do so, we analyze health information sharing within the concept of the PANACEA project that facilitates the design, implementation, and deployment of a relevant platform. The research presented in this paper provides evidence and argumentation toward advanced and novel implementation strategies for a state-of-the-art information sharing environment; a description of high-level requirements for the transfer of data between different health-care organizations or cross-border; technologies to support the secure interconnectivity and trust between information technology (IT) systems participating in a sharing-data “community”; standards, guidelines, and interoperability specifications for implementing a common understanding and integration in the sharing of clinical information; and the use of cloud computing and prospectively more advanced technologies such as blockchain. The technologies described and the possible implementation approaches are presented in the design of an innovative secure information sharing platform in the health-care domain.

## Introduction

Information technology (IT) has long been identified as a cornerstone for the efficient, costless, timely, and reliable health-care delivery (1, 2). The availability of health-care information and patient records in digital form facilitates the persistence and posterity of valuable information and greatly support the decision-making process and even the extraction of new knowledge at both the individual and population levels. In our previous work, we have emphasized on current state of the art about cyber security in the health-care domain with emphasis on current threats and methodologies (3). This work is paraphrasing the famous words by John Donne, “no IT system is an island, entire of itself.” Today, in a highly connected world where geographic boundaries have been largely eliminated and people can freely move between cities, states, countries, or continents, the requirement for two different information systems to exchange a person's clinical data or medical history becomes vital and persistent. Sharing health information [or health information exchange (HIE)] through electronic means greatly improves the cost, quality, and patient experience of the health-care delivery.

**Figure 1:**
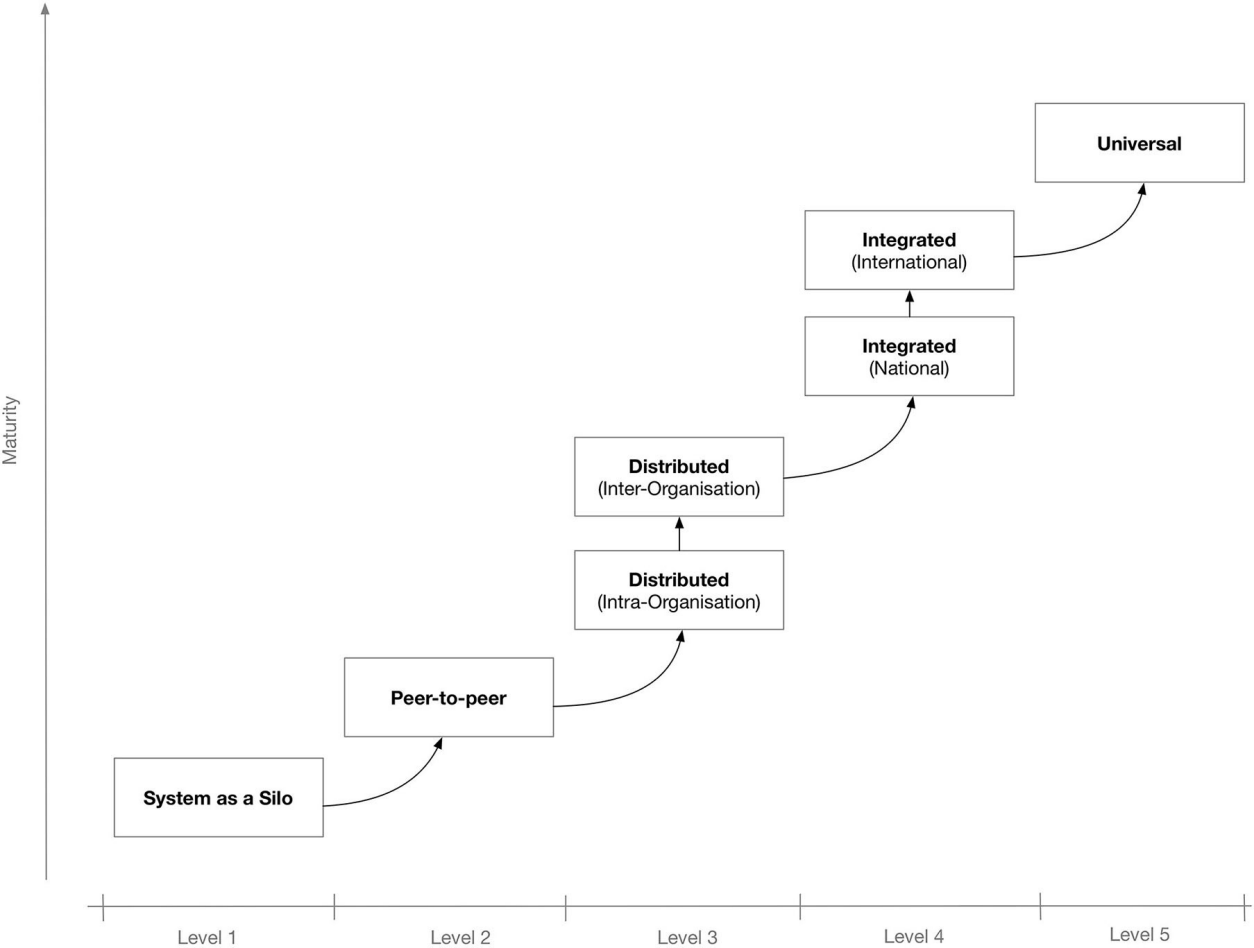
A maturity model for interoperability in eHealth [adapted from (4)].

To better secure the IT system's potential for interconnectivity and cooperation with other systems, the use of interoperable technologies and standards is needed. Depending on the extent and scope of the envisaged shared information spaces, there may be different levels of interoperability. Figure 1 shows a proposed “maturity” model for interoperability in eHealth (4). The model consists of five levels that, incrementally, describe a more mature version of an interoperable infrastructure, starting from Level 1 for non-connected eHealth applications; Level 2 where a single eHealth application is directly linked to another application for simple data exchange (5); Level 3 for distributed systems that agree on protocols used, data formats, message exchange patterns (6), etc.; Level 4, where eHealth applications from different suppliers that serve a common goal are linked but the applications do not need to have common objectives (7); and finally, at the “universal” Level 5, where diverse eHealth applications connect to an open, interoperable infrastructure possibly spanning multiple countries (8, 9).

Interoperability and data sharing in the health-care domain is additionally challenging due to the multiplicity of the stakeholders, that is, the entities that operate (or are involved in any way) in this domain and which will be affected by any “disruption” or reform of the system. Some of the most important stakeholders or actors are therefore the following:

- The patients who are actually treated or, in general, are the recipients of the health services.
- The medical professionals (physicians and medical personnel) to provide the medical care.
- The health-care organizations (HCOs) (health-care providers) as represented by their director boards who actually administer the health delivery from a business perspective.
- The insurance companies that provide health coverage plans.
- The pharmaceutical companies that produce and market medications to be prescribed by physicians for the treatment of patients.
- The governments and other regulatory parties who control, coordinate, and set the rules, rights, and obligations of any involved party.

All these actors could have an influence in the design of a data sharing system and can also set important, and conflicting in some cases, requirements. For example, patients would like to have their medical record shared but only after their approval and only with specific authorized personnel in specific circumstances; an HCO can be extremely cautious about sharing the data of their patients with another organization because they are concerned by the security and availability of their systems; governments of EU member states (MSs) can impose strict laws about the transfer of their citizens in cross-border health-care treatment scenarios; and medical professionals require fast and effortless access to a patient's medical history in emergency situations, which cannot be the case if time-consuming authorization processes are the norm. It is imperative, therefore, even though the objective is to design a technical solution for the sharing of clinical data, that all these constraints and requirements are considered and addressed in a satisfying manner.

From a strictly technical point of view, the sharing platform may need to interoperate with a large number and diverse set of IT systems, each with their own protocols, data formats, etc., Some of the most important systems that manage patient-related data and could be used as data sources for information sharing are as follows:

- Electronic health records (EHRs): These are patient-centered systems that store and manage clinical information, such as, a patient's medical history, diagnoses, medications, immunization dates, allergies, radiology images, and lab and test results. They are managed by authorized personnel, usually in the context of a single HCO, although they can span more.
- Personal health records (PHRs): These are electronic applications that are used by people managing their own health information in a private and confidential environment. They are simpler systems than EHRs, and in some cases, they can be connected (temporarily or otherwise) to more enterprise level HCO systems (e.g., EHRs or other hospital information systems).
- Laboratory information systems (LISs): Used inside hospitals and clinics to record, manage, and store data for clinical laboratories in a patient-centric way (sending laboratory test orders to lab instruments, tracking those orders, and then recording the results in a searchable database).
- Picture archiving and communication system (PACS): These are systems used in a clinical setting for the storage and convenient access to medical images from multiple modalities (source machine types). Digital Imaging and Communications in Medicine (DICOM) is the standard format and suite of protocols for the storage and transfer of images from PACS services.

Moreover, in a health ecosystem, there may be additional systems, for example, for the management of insurance claims and clinical decision support systems (CDSSs).

**Figure 2:**
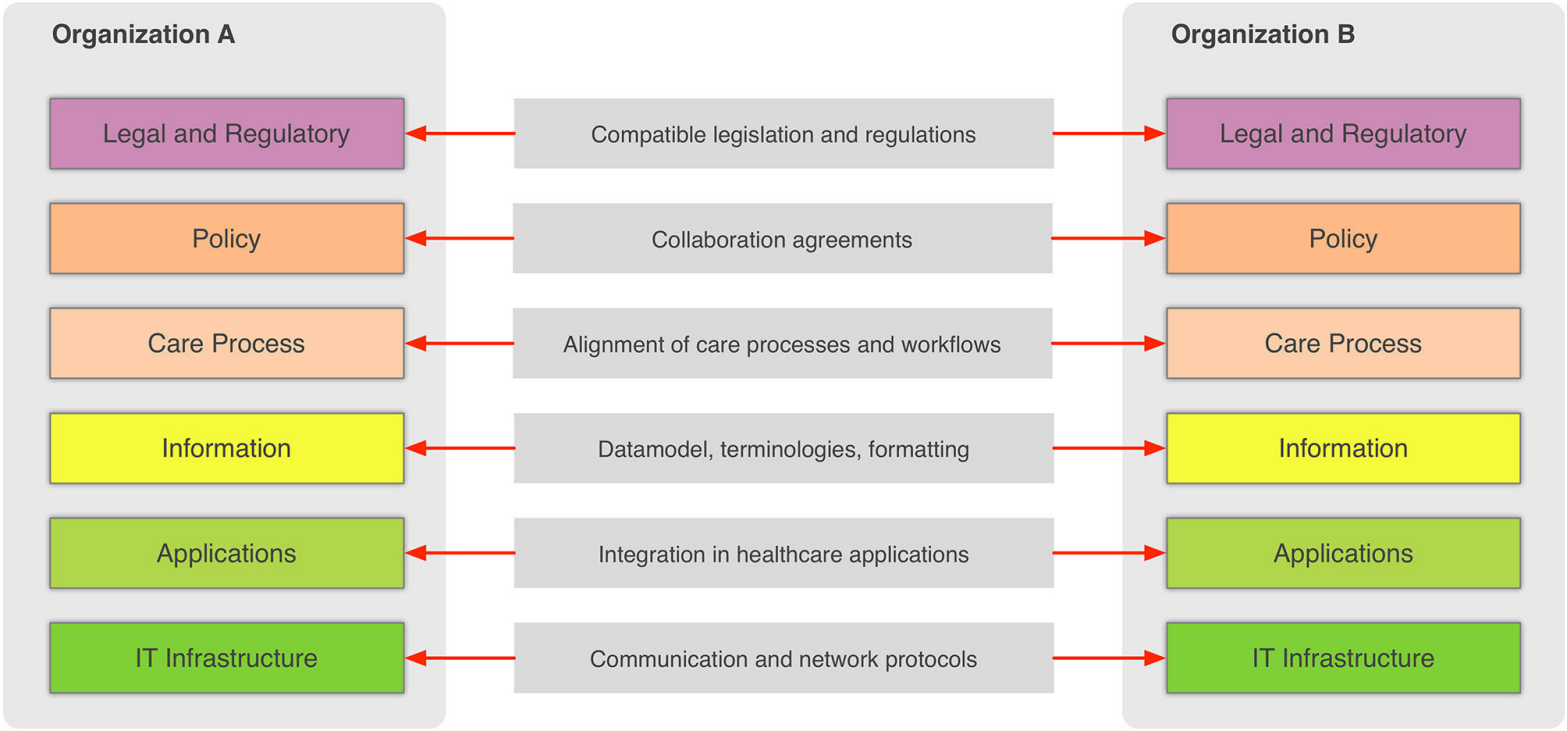
Alignment of interoperability levels between two communicating organizations.

In the general context, information sharing involves more than one party (health-care providers, organizations, etc.,) that needs to cooperate and agree on the way the exchange of information happens, and what the rules and policies are that govern it. Interoperability involves many different aspects, such as legislation and guidelines, contracts, and agreements between exchanging parties, governance and maintenance, shareable workflows, standardized data elements, semantic and syntactic choices, applications, technical infrastructure, and safety and privacy issues. The Refined eHealth European Interoperability Framework (EIF) is a set of recommendations that specify the standards, protocols, procedures, and policies that when deployed can improve the interoperability of eHealth applications within the EU and across its MSs by providing specific recommendations for all these aspects (10). Figure 2 depicts how these aspects can be represented in interoperability “levels” that permit two different organizations to communicate.

This framework provides a great overview of the needed “glue” so that two or more health-care environments can collaborate and serve as a common, multilevel, and multi-perspective model on the interoperability requirements. The six different levels of the (refined) EIF are the following:

- Legal and regulatory: Legislation and regulatory guidelines that define the boundaries for interoperability across borders, but also within a country or region.
- Policy, which represents the contracts and agreements between the sharing organizations so that trust is established and responsibilities are assigned.
- Care process: Shared workflows that define how the integrated care is delivered and how these workflows are managed.
- Information, which defines the data models, the concepts and their values, the terminologies, and controlled vocabularies that cater for the common understanding of the exchanged electronic messages.
- Applications, which define how the data are extracted from and imported to the health-care information systems and how the transport of the data takes place using health-specific technologies and standards.
- IT infrastructure is at the lowest level and corresponds to general-purpose communication and network protocols.

This generic eHealth Interoperability Framework is highly relevant for the use cases of the health information sharing since sharing is greatly facilitated between interoperable systems/organizations. Here, we focus on the sharing of health-related information across HCOs and even across countries and continents. This is an important use case to improve the secure and efficient delivery of health care across Europe (11, 12). Cross-border health care in Europe has been recognized as of 2011 with Directive 2011/24/EU, which established patients' rights to access safe and high-quality health care, including across national borders within the EU, and their right to be reimbursed for such health care (13). As can be deduced by considering the Refined eHealth EIF (Figure 2), the cross-border sharing of clinical information is a complex scenario due to the fact that data need to be transferred between different countries and therefore, requires overcoming barriers such as, establishing a common trust framework, uniquely identifying citizens, and translating between different schemas and terminologies. This paper presents current approaches to address most of these issues in the European context while presenting and evaluating emerging technologies such as blockchain that have been in the limelight recently. Our objective is to take advantage of both well-established and novel technologies that complement each other in order to design an architecture for the secure exchange of clinical information in the European context.

## Materials and Methods

### Current Status on Standards-Based Health Data Exchange

In the health-care industry, large standards developing organizations have defined numerous standards, data formats, terminologies, etc., in order to support the design and building of interoperable IT systems. Perhaps the most well-known and most important standards are the ones introduced by Health Level 7 (HL7) and SNOMED, which can be used as a foundation for the development of data exchange standards among eHealth systems (14). Two more standards organizations are Integrating the Health Enterprise (IHE), which focuses primarily on integration and interoperability; and the Clinical Data Interchange Standards Consortium (CDISC). CDISC produced the Operational Data Model (ODM) to “facilitate the regulatory-compliant acquisition, archive and interchange of metadata and data for clinical research studies” (15). ODM is an XML-based format that provides a number of constructs for modeling electronic case report forms (CRFs) and can also be used in sending forms data from a clinical trial system to an EHR system. In the area of medical devices, the Continua Health Alliance, a non-profit, open-industry coalition of health-care and technology companies working to establish a system of interoperable personal health solutions, develops an ecosystem of connected technologies, devices, and services that will enable the more efficient exchange of fitness, health, and wellness information (16). Among its proposed standards, Continua proposes specifications and standards such as Bluetooth, USB, medical devices (IEEE 1173), and HL7 to enable people to use home-based devices to monitor their weight, blood pressure, and glucose and blood oxygen levels and to share these data with their health-care professionals.

The exchanged information can be in multiple data formats based on the type of data, device category, etc., (17). Some of the most common formats based on the health applications using them are the following:

- For medical imaging, the use of DICOM is almost universal and defines not only the content (DICOM file format) but also communication protocols for the exchange of medical images (18).
- In the area of DNA sequencing and other -omics data formats including FASTQ file format (19), which is used to store sequence information, and the standard flowgram format (SFF), which is used to encode sequence reads (20).
- The majority of the EHR systems adopt the HL7 standard clinical document architecture (CDA) as the interoperable data format (21). CDA is part of the HL7 version 3 family, and it is based on a reference information model (RIM) that serves as a semantic model that consists of a set of structural components (e.g., classes with data types) and semantic relations that are used to represent clinical notes in the form of an extensible markup language document.

The use of controlled vocabularies and terminologies allows for the unambiguous representation of important value sets, such as, the diagnosis and the medicines (22). The following are examples of such terminologies:

- The International Classification of Diseases (ICD) provides a common language for reporting and monitoring diseases, used throughout the world to compare and share data in a consistent standard way between hospitals, regions, and countries and over periods of time. It is used to classify diseases and other problems for payment, management, and research, as recorded on many types of health records including medical records and death certificates. ICD-11 is the latest version of it, whereas ICD-10 (released in 1993) remains widely used.
- SNOMED CT, already mentioned above, is the most comprehensive multilingual clinical health-care terminology available. It is used in EHR systems to facilitate clinical documentation and reporting and to retrieve and analyze clinical data. SNOMED CT is both a coding scheme, identifying concepts and terms, and a multidimensional classification, enabling concepts to be related to each other, grouped, and analyzed according to different criteria.
- Logical observation identifiers names and codes (LOINC) provides a set of universal identifiers for medical laboratory observations. LOINC provides codes for the observation names (e.g., eye color), not the observation finding (e.g., blue eyes). LOINC therefore provides codes for questions; and where needed, other vocabularies, such as, SNOMED CT, provide codes for the answers.
- The Unified Medical Language System (UMLS) is an important terminology resource, intended for use mainly by developers of health information systems. The UMLS “Metathesaurus” uses several different source vocabularies and seeks to reflect and preserve the meanings of concept names and relationships from these sources. It is therefore a valuable resource for the translation between the different source vocabularies.

Application-level interfaces are also needed to support the communication and exchange of the standards-based encoded information. The role of HL7 is principal on this front: HL7's name comes from “Level Seven,” which, according to the Open Systems Interconnection (OSI) model that standardizes communication functionality in IT, corresponds to *application layer*. From its establishment in the late 1980s, HL7 was therefore focused on exchanging information within hospitals. The focus remains almost the same today, but HL7 has progressed from different paradigms over the years, in order to describe the structure, semantics, and management of the exchanged information. The development of HL7 version 3 (HL7v3) started around 1995 in order to introduce more consistency between the implementations of version 2 following an object-oriented development methodology. The most recent proposal by HL7 is Fast Healthcare Interoperability Resources (FHIR), which leverages web technologies to overcome the complexity of HL7v3 (23).

On the other hand, the IHE initiative has defined a number of “integration profiles,” which are detailed specifications for communication among systems to address key clinical use cases, all based on established standards. IHE profiles organize and leverage the integration capabilities that can be achieved by coordinated implementation of communication standards, such as, DICOM, HL7, W3C, and security standards (24). Some of the IHE integration profiles that might be interesting in the context of interfacing health information systems and sharing of clinical information are as follows:

- Cross-Enterprise Document Sharing (XDS): Share and discover EHR documents between health-care enterprises, physician offices, and clinics, acute care in-patient facilities, and PHRs.
- Patient Demographics Query (PDQ): Enables applications to query by patient demographics (e.g., name) for patient identity from a central patient information server.
- Patient Identifier Cross Referencing (PIX): Allows applications to query for patient identity cross-references between hospitals, sites, HIE networks, etc.,
- PDQ HL7 v3 (PDQv3): Extends the PDQ profile leveraging HL7 version 3.
- PIX: Extends the Patient Identifier Cross-Reference profile leveraging HL7 version 3.
- Cross-Community Access (XCA): Allows to query and retrieve patients' EHRs held by other communities.
- Cross-Enterprise Document Reliable Interchange (XDR): Exchanges health documents between health enterprises using a web service-based point-to-point push network communication.
- And many others.

There are two main architectural approaches for the implementation of an information sharing platform: centralized and federated (25). In the centralized approach, a central data warehouse and accompanied services act as middlemen for the exchange of information and a single source of patient data that are shared among the participating organizations. On the other hand, in the federated architecture, a central infrastructure is also in place, but in this case, it merely acts as a facilitator for locating the data sources. An example of this case would be a common registry that stores only the links to the original patient records, medical images, etc., while the linked data are not transferred outside their primary premises unless explicitly requested by any interested client system. In addition to these opposite approaches for designing a distributed information sharing platform, there are also various hybrid options, such as using messaging with “publish-subscribe” communication that can be introduced to complement either the centralized or federated architectures.

There are advantages and disadvantages in all of the abovementioned deployment options. For example, in the federated approach, there are more strong concerns about the privacy, security, and availability of the data shared and their original sources (26). The operation of a mission-critical radiological information system (RIS) in a hospital can be severely affected if multiple peers request DICOM images from its PACS, and this poses an additional burden and cost for the acquisition and management of adequate infrastructure in the source organization. Instead, a centralized strategy allows for easy access to the whole information shared but also leads to a concentration of the costs for maintaining the infrastructure needed and can be problematic at the operation level (a “single point of failure”). Furthermore, there are more costs on integrating the different data sets under a common “schema,” resolving conflicts or even supporting the timely update of the persisted information when a source system acquires new or modified data.

### Emerging Supportive Technologies: Blockchain

Blockchain is a *decentralized, distributed* data structure used to store *transactions* (aggregated in blocks) across many computers (27, 28). Blockchain has been extensively used for Bitcoin (29). For health care, we have seen the work of Kuo et al. (30) where they performed a systematic review on how blockchain can be used in health-care applications. Blockchain core is the embedded distributed ledger technology able to support for data integrity, authenticity, and origin. In blockchain, each block is linked to the previous one through a cryptographic hash, and it is a data structure that allows to store a list of transactions (31). In the blockchain, a transaction abstracts and allows to keep track of an exchange or interaction between two entities. Transactions are created and exchanged by peers of the blockchain network and modify the state of the blockchain data structure. An efficient categorization and a comprehensive overview of the latest privacy-preserving mechanisms and policies regarding privacy-preserving methods and characteristics, in smart electric grids, focusing on the use of the blockchain technology and the multi-authority access control paradigm is studied in (32, 33).

Concerning data access, we can have the following:

- *Public blockchain*: There are no restrictions on reading blockchain data and submitting transactions for inclusion into the blockchain.
- *Private blockchain*: Direct access to blockchain data and submitting transactions is limited to a predefined list of entities.

Concerning data management, we can have the following:

- Permissioned blockchain: Transaction processing is performed by a predefined list of peers with known identities.
- Permission-less blockchain: No restrictions on identities of transaction processors (i.e., blocks creators).

**Figure 3:**
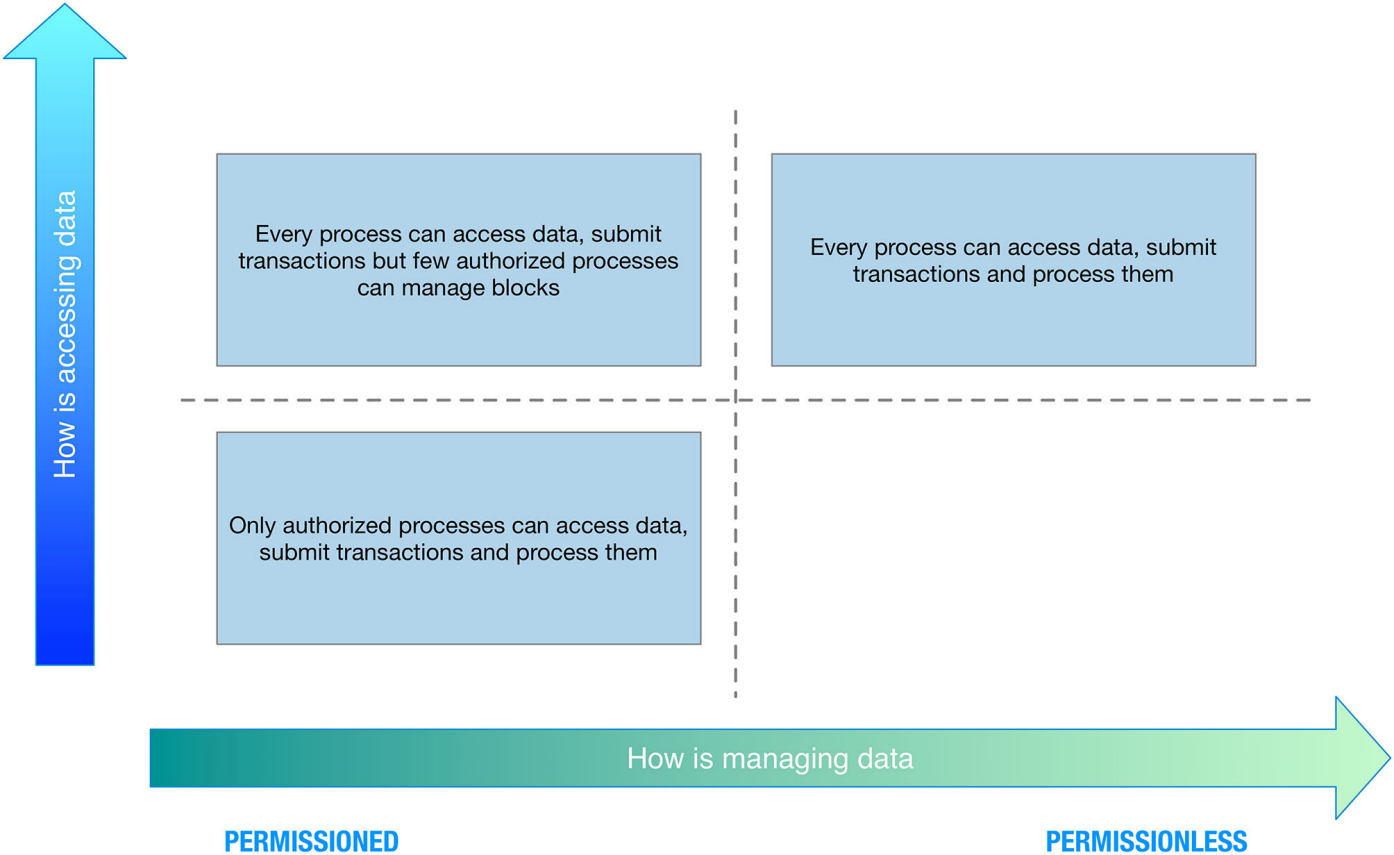
Blockchain classification overview.

Combining the two perspectives, we can have four categories as depicted in Figure 3.

Blockchain supports auditability and transparency, as any reader is able to verify the correctness of the state of the system. Indeed, by storing all the transactions, it is possible to re-play (starting from a correct checkpoint) the entire history and check that the current state is consistent with the set of recorded transactions. It is important to note that, when using a blockchain to store data, there exists an inherent trade-off between transparency and privacy. Indeed, if the primary requirement is to have a fully transparent system, we need to accept that anyone is allowed to see any piece of information (sacrificing privacy). Conversely, if the primary requirement is to have a private system, it will not provide transparency. A trade-off between transparency and privacy is however possible, but it will come at the cost of efficiency, as it would require employing complex cryptographic primitives.

## Results and Discussion

### A Novel View on Information Sharing

Nowadays, the sharing of a patient's clinical information between two HCOs (e.g., hospitals) usually requires a great amount of manual work in order to check and validate patient's consent (by consulting signed papers) or, at worst, results in privacy loss by extending the trust circle, e.g., to all physicians from the requesting organization. The main limitation of the current sharing pattern can be summarized in the following:

1. Sharing medical data may require time and possibly multiple interactions, also involving the patient in the loop.
2. Sharing medical data is currently a physical point-to-point interaction. If the same set of data needs to be shared with multiple parties, it would require multiple sharing patterns to be in place.
3. Sharing is currently asymmetric. Organization A may have the consent to share patient's data with organization B, but the inverse may not be true.

**Figure 4:**
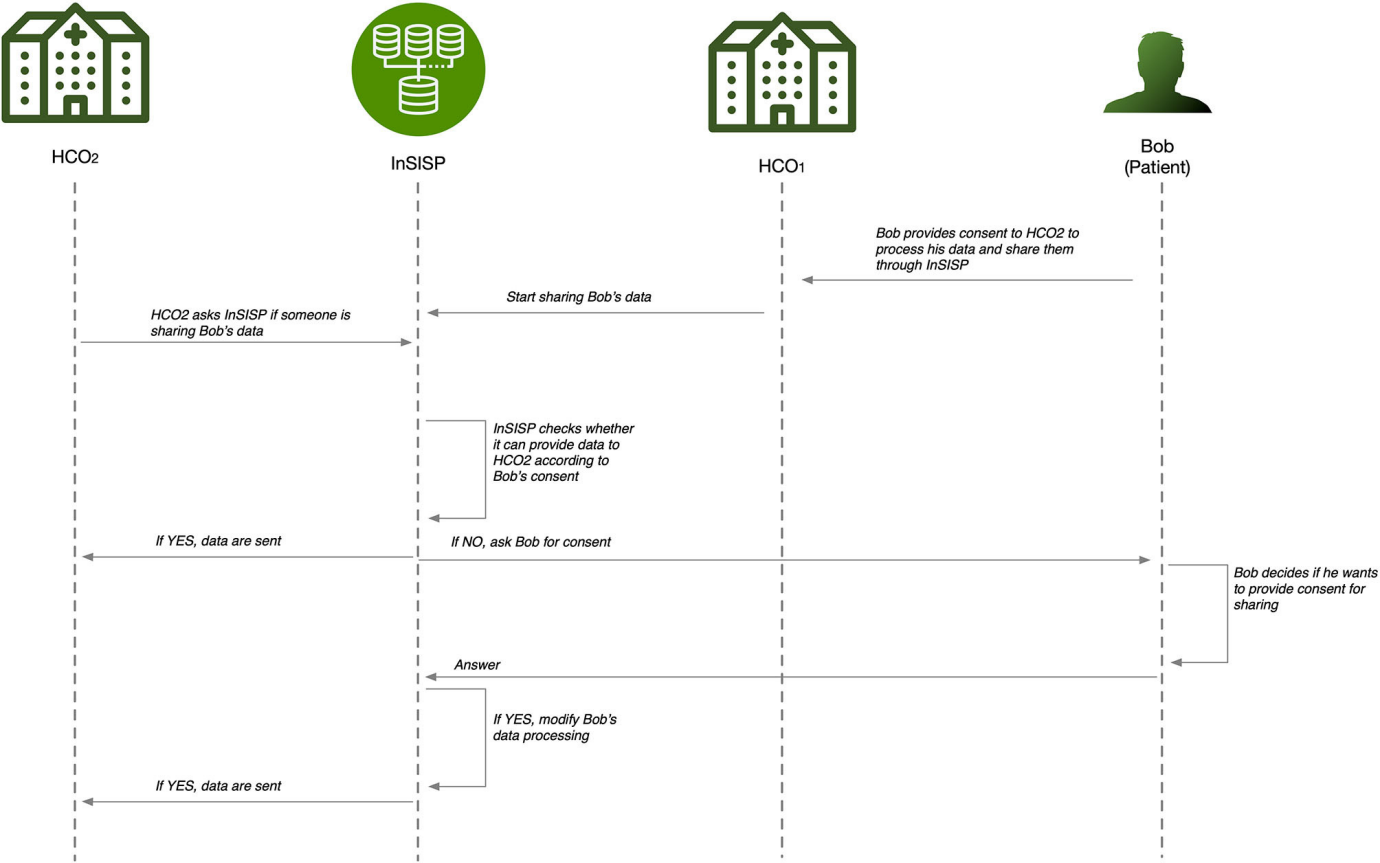
InSISP sharing pattern for medical data.

In order to overcome these deficiencies, we aim to design a platform—the Innovative Secure Information Sharing Platform (InSISP)—that is able to support a fast and efficient medical information sharing at both national and cross-national levels, taking into account sharing constraints, included those imposed by the General Data Protection Regulation (GDPR). It is imperative that patient's consent is central in this framework, and one of the challenges is to make the consent management robust, simple, and secure. A sequence diagram of the possible interactions to support the data sharing is shown Figure 4.

**Figure 5:**
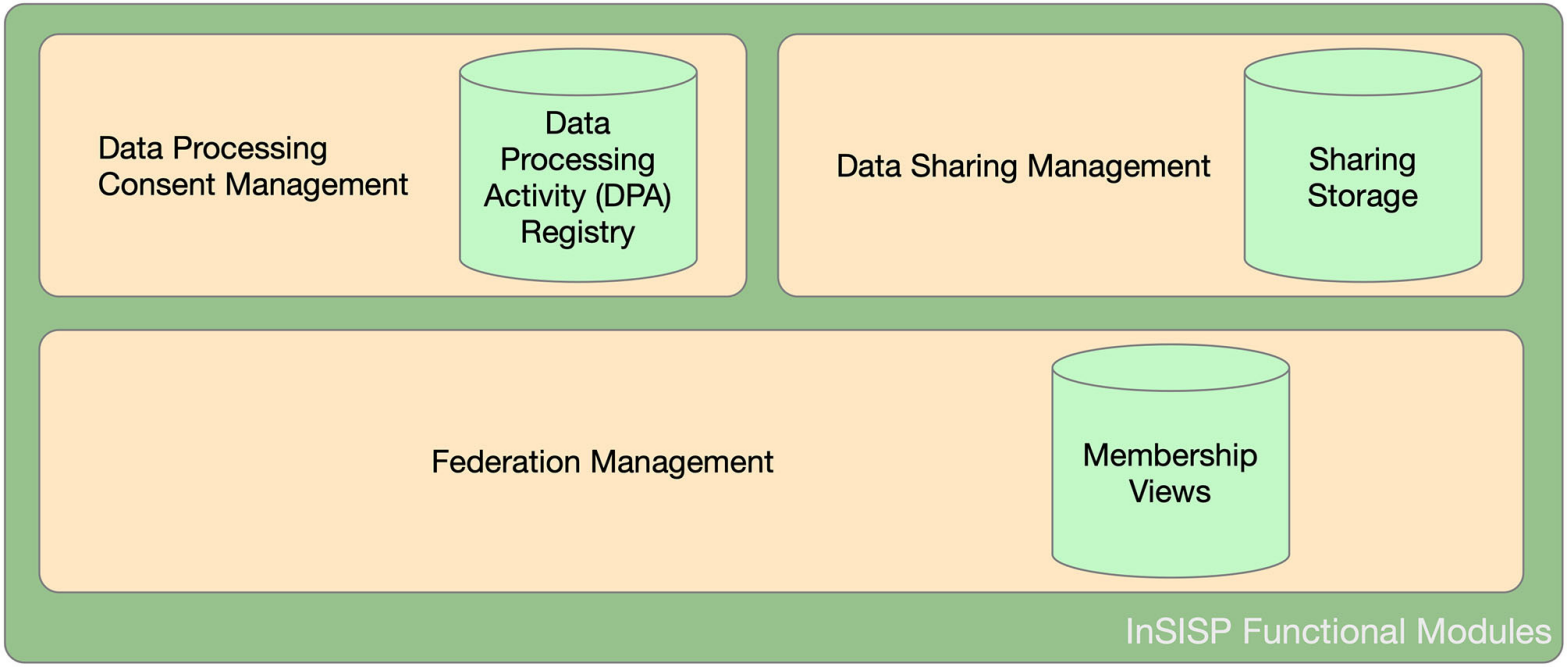
InSISP functional decomposition.

From an abstract point of view, InSISP can be seen as a data repository that can be accessed by HCOs (i.e., clients) to store and retrieve shared data by using a common interface and format (e.g., CDA Release 2 using standard vocabularies such as SNOMED and LOINC). The shared data repository is surrounded by a federation of collaborating entities (organizations/clients) that is dynamic and evolving. Once the federation is established, participating entities can start sharing data according to the data processing consent provided by patients. To this aim, we can identify two additional functionalities: (i) data sharing and (ii) data processing consent management. Figure 5 summarizes a possible decomposition of the InSISP and highlights the three storage components.

#### Federation Management

The federation management functionality has the aim to manage the federation life cycle, and in particular, it should allow new HCOs to join and HCOs no longer interested in participating in the federation to leave. In particular, this functionality supports the following operations:

1. *Join the federation*: HCOs should be able to become part of the federation at any time. When considering a membership service, all the members are assumed to be uniquely identified. So from the perspective of the InSISP development, there should be an external service that is able to support the identification of members and to provide them with a digital identity.
2. *Leave the federation*: HCOs may decide to leave the federation at their will, and the InSISP should support the removal of the entity from the membership and should notify the end of the sharing to connected entities.
3. *Get the federation membership*: Allows an HCO participating in the federation to get the current membership and know the set of HCOs potentially involved in the sharing, including their identification, public keys, and the categories of data that are currently available for the sharing.

The federation management intrinsically relies on the execution of a distributed protocol running, and thus, there are two main options to implement a federation membership service: (i) *client/server* or (ii) *peer to peer*.

In the client/server case, the current membership of the system is maintained by a trusted third party (TTP). When a new HCO wants to join the federation, it simply needs to contact the TTP and identify and authenticate itself with the TTP that will proceed by adding it to the current view. Similarly, when an HCO in the federation wants to leave, it simply needs to notify the TTP that will remove it from the current view. The current membership can be obtained again by querying the TTP. The main advantage of this option is that all the complexities of the membership management are delegated to the TTP. However, this also implies that the TTP is clearly a single point of failure for the system as well as its main bottleneck.

In the peer-to-peer approach, HCOs collaborate to maintain a consistent view of the system by exchanging messages and trying to reach a consensus on the sequence of views generated to include new members and to remove old ones.

Chockler et al. (34) discussed in detail the formalization and the specification of the group membership service, while more recently, Aguilera et al. (35) considered the problem of building a reconfiguration service to support the development of a distributed shared storage.

Let us note that in all these cases, the emphasis is on how to provide a consistent view to all the members. To the best of our knowledge, there does not exist any approach investigating the cost of realizing a membership service using blockchain technologies.

The main advantage of the peer-to-peer approach is its intrinsic resilience. In addition, in peer-to-peer settings, it is also possible to consider a blockchain-based approach to construct the sequence of consistent views providing the view auditability property for free. The main drawback is the cost imposed by the management of consistent views, as it requires to run coordination and synchronization protocols among all the participants that would bring poor scalability in case of a highly dynamic federation.

#### Data Processing Consent Management

The data processing consent management function has the aim to support the development of a digital data processing activity registry to store and access patients' data processing consent. The data processing consent is granted by a patient for a specific set of data to a specific set of entities and for a specific purpose and period of time. In order to support the automatic verification of patients' consent, such information must be stored and managed by the HCO in an electronic form (e-Consent) (36–38). Also, every patient has the right to modify his/her consent and has the right to be forgotten; i.e., at any point in time, he/she may ask to revoke all his/her previous consent (while also erasing any data identifying him/her).

To this aim, the data processing consent management functionality should offer the following operations:

1. *Provide new consent*: It allows to add a new entry to the table and to specify the beneficiary entities and to set the expiration time of the consent.
2. *Update consent*: It allows to modify existing consent by allowing the sharing with a new beneficiary or by removing a beneficiary.
3. *Remove (all) consent*: It basically implements the right to be forgotten by deleting all the consent previously provided by a specific patient.

##### Discussion About Possible Design and Deployment Options

Let us note that the data processing consent management function supports every HCO in managing its own data processing activity registry. Thus, from this point of view, we can say that it is local to every HCO.

As a consequence, the most appropriate choice is to design it as a local data store managed and accessed only by one HCO. Of course, in order to increase the resiliency and security of the storage, it can be also replicated, but all the replicas will still be managed by the same HCO.

A distributed design raises a privacy issue in patients' information. Indeed, even if data in the registry are not sensible by themselves, they could be easily correlated to infer sensitive information about patients and would result in a privacy violation; e.g., by looking to the list of HCOs where Bob did his analysis, you may infer that Bob is affected by a specific disease. To solve this issue, it is necessary to employ anonymization scheme generation an extra cost without any particular advantage in terms of reliability or security.

#### Data Sharing Management

Data sharing is the core functionality of the InSISP, as it manages the real transfer of medical data between parties. It offers just one main operation, i.e., the Get Data, which is used to retrieve a specific piece of data for a specific patient and transfer it according with the patient's consent.

We can consider three main options to design and deploy this functionality:

1. Client/server.
2. Peer to peer with message exchanges.
3. Peer to peer with shared memories.

**Figure 6:**
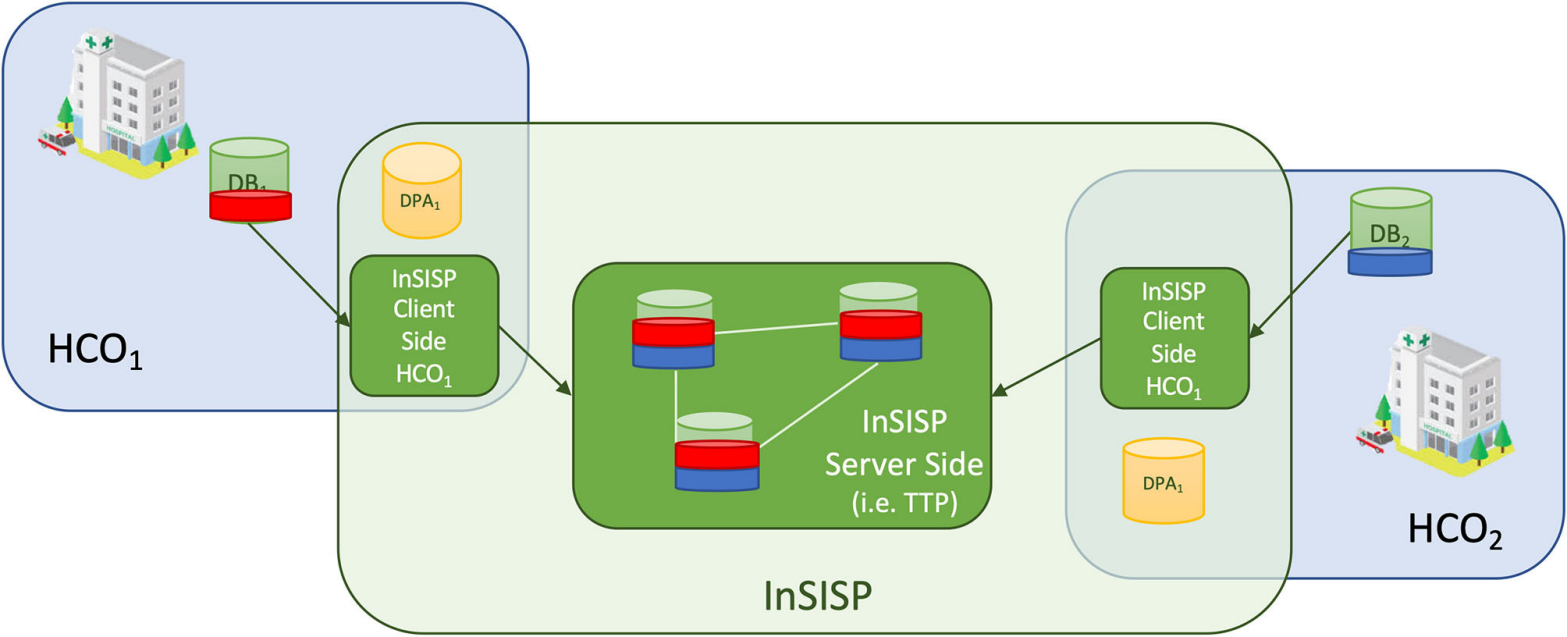
Overview of the client/server design with a trusted third party (TTP).

In the client/server case, data available for the sharing are copied and pushed toward a centralized TTP that will take care of satisfying the sharing request. In order to be GDPR compliant, a specific consent to move data to the TTP must be signed as well as the consent to share data with all the federation members^1^. Figure 6 shows an overview of a possible client/server design.

In the peer-to-peer case, the idea is that the sharing is realized by letting HCOs in the federation cooperate with each other. We can distinguish two cases: cooperation realized through message exchange and cooperation realized through shared memory.

**Figure 7:**
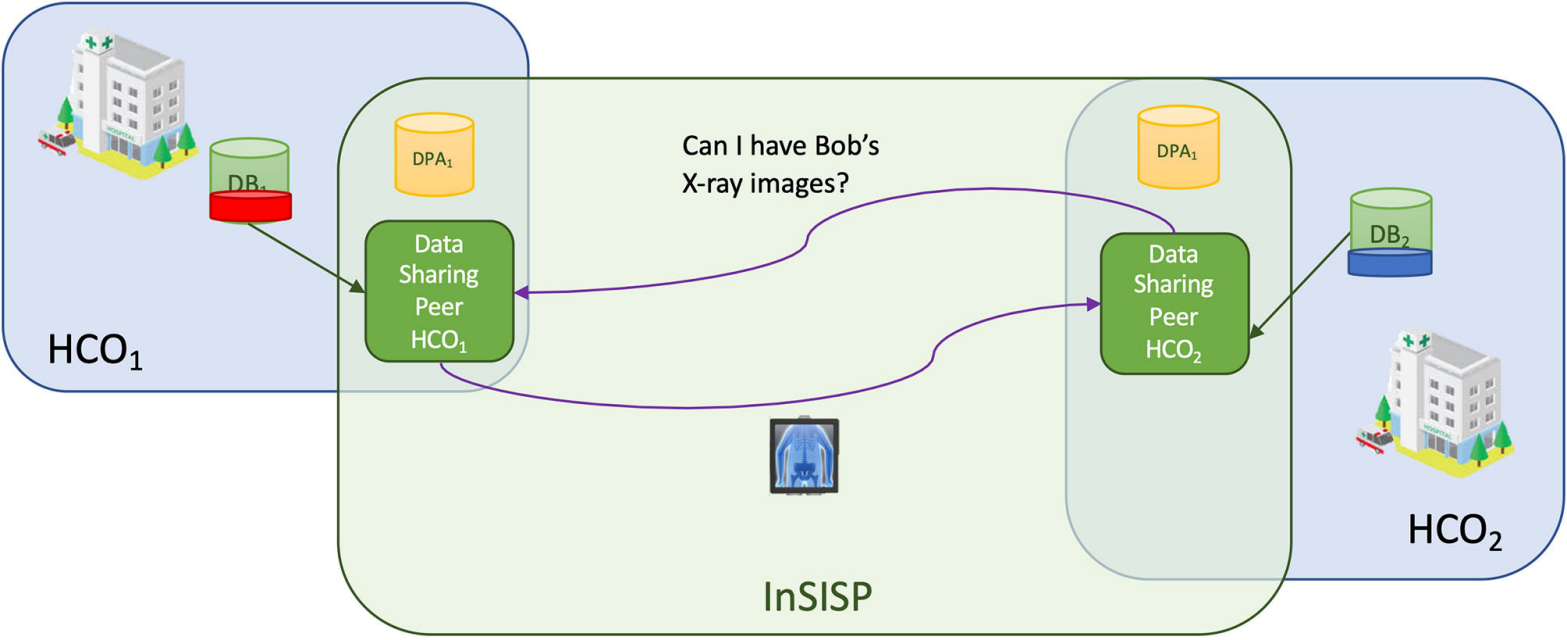
Overview of the peer to peer with message exchange design.

In the message exchange case, the sharing is realized using an *ad hoc* request–reply communication pattern as shown in Figure 7.

**Figure 8:**
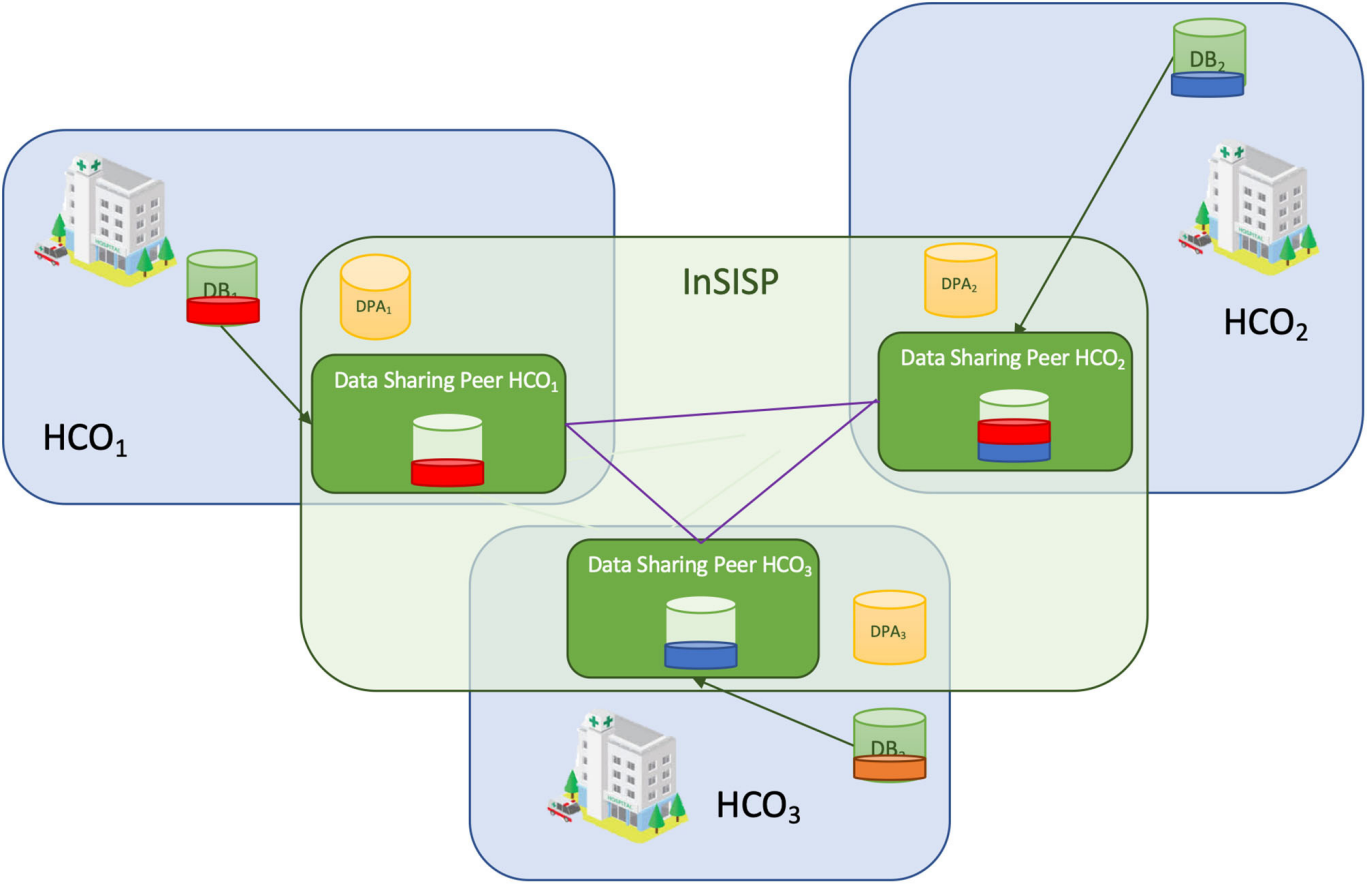
Overview of the peer to peer with shared memory design.

In the peer to peer with shared memory design (Figure 8), each HCO creates a shared memory space where it stores all the pieces of data that can be shared according to the data processing activity registry. As an example, let us consider the case where Bob provided the consent to HCO_1_ to share his X-ray images with HCO_2_. This means that HCO_1_ and HCO_2_ create locally a shared space where they will store a copy of all Bob's X-ray images (the red slice in Figure 8).

##### Discussion About Possible Design and Deployment Options

Technically, all the three considered designs are feasible. However, as anticipated in the previous section, the client/server scenario poses several challenges from the point of view of the consent needed in order to make it compliant, and in particular, the main issue is the large set of consent that is necessary and that patients may be reluctant to provide. Instead, the two point-to-point designs solve this issue, as they exploit locally the consent information and move data only toward authorized HCOs.

#### Summary of Recommendations

According to the considerations done in the previous sections, Table 1 summarizes the viable options for the design of each functionality of the InSISP.

**Table 1:** Summary of design options and recommendations.

		**Is it a valid design and deployment option?**	
		**Yes**	**No**
DPA registry supporting the data processing consent management	Local	X	
	Centralized		X
	Distributed		X
Sharing storage supporting the data sharing	Local	X (peer to peer with message exchange)	
	Centralized		X
	Distributed	X	
Membership view supporting the federation management	Local		NA
	Centralized	X	
	Distributed	X	

*DPA, data processing activity*.

### Evaluation of Emerging Technical Solutions: Blockchain

In order to evaluate if blockchain is a valid option to support the InSISP deployment, the methodology presented in (39) considers the following three steps:

1. Requirement analysis to assess blockchain benefit.
2. Evaluation of the most appropriate blockchain solution where the designer is guided on the choice of the most suitable blockchain category, based on blockchain-specific criteria depending on who are readers and writers of the data and who is allowed to generate data.
3. Blockchain configuration selection, which assists the designer throughout the decision-making process for the configuration of the blockchain compliantly with the chosen category and the given project requirements.

Let us remark that blockchain is intrinsically a distributed system, and it makes no sense trying to use it when a distributed setting is not appropriate, which means that it can be used for the federation membership and for the data sharing functionalities of InSISP. In the following, we will evaluate the suitability of blockchain-based solution by adoption of the methodology described in (35).

In fact, trying to evaluate the blockchain technologies for the data sharing scenarios and design solutions described above, the first step is the analysis of requirements related to the component under analysis in order to understand the benefit of adopting a blockchain-based solution for its low-level design and development.

The factors that are considered in this step are listed in the following:

- Data or state storage. The first element to consider is to check if the module under analysis needs to store data or system state. If no information needs to be stored, clearly no blockchain is needed.
- Immutability and data integrity. With immutability, we refer to the property of a data to never change (i.e., a constant value that is never updated). If immutability is a requirement, then blockchain is certainly an option, as this is probably the most distinctive property of any blockchain. Integrity is strictly related to immutability, and this is why they are analyzed together, also considering that they are both closely related to cryptography. If a component requires data protection from unauthorized modifications, then this requirement can be met with a blockchain.
- Non-repudiation. Non-repudiation means that the author of some message/data cannot deny that it produced the message. This is another fundamental property that can be easily satisfied using blockchains.
- Multiple writers. This criterion considers the multiplicity of entities in charge of writing data in the storage. If only one entity is a writer, thus a common database is probably most appropriate than a blockchain especially from the performance perspective, i.e., in terms of throughput and latency.
- TTP always online. A TTP is an entity that facilitates interactions between mutually mistrusting entities. If in the system a TTP is required and it is planned to be always online, entities can delegate to it write operations as transactions, or state changes. Therefore, the TTP plays the role of a trusted deliverer and verifier. In this case, a blockchain, known for being a trust less technology, becomes useless, and the methodology brings to the related output. Otherwise, it can happen that the involvement of a TTP is planned but not for being always online: in this case, it could play the role of an authority giving authorizations for permissioned blockchains. Alternatively, a TTP may not exist at all. In the latter situations, it is not possible to exclude the recommendation of using a blockchain.
- Writers are known and trusted. If all the entities interested in writing know and mutually trust each other, a blockchain is superfluous and not recommended (again mainly for performance issues).

**Figure 9:**
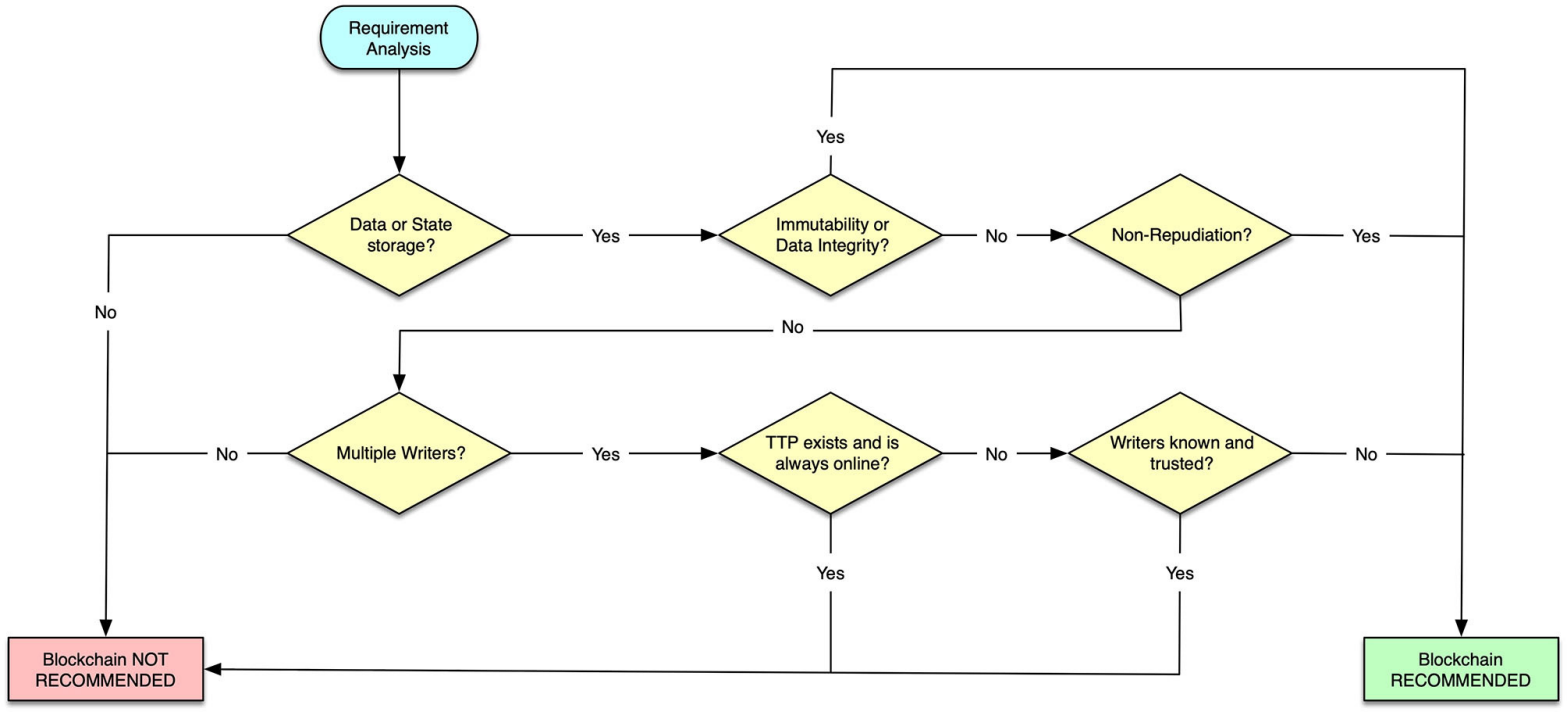
Flowchart: blockchain, yes or no? adapted from (39).

The flowchart of this analysis step is shown in Figure 9. According to this decision flowchart, Tables 2, 3 answer the relevant questions for the assessment of blockchain in the federation management and data sharing operations. Following the flowchart in Figure 1 at a first glance, we get that blockchain is not recommended to support the implementation of the federation membership mainly because there exists a basic level of trust between members of the federation. In addition, we are also assuming that the federation membership is also relying on a trusted external service providing digital and secure identities to participants. However, we also highlighted the opportunity to consider a non-repudiation requirement, and we considered the importance of preserving data integrity (i.e., to ensure that the current membership cannot be altered). Thus, if these two requirements become more relevant or if assumptions on the identity platform or about trustworthiness of participant cannot be met, then blockchain becomes immediately a viable solution.

**Table 2:** Assessment of blockchain in the federation management.

**FEDERATION MANAGEMENT**	
**Do you need to store data or state?**	**YES. The data to be stored are represented by the identifiers of federation members and their related information (e.g., keys used to verify the integrity of the messages)**.
**Do you have immutability or data integrity requirements?**	**NO. HCO identifiers should not change. However, the set of identifiers may change in time, as well as some of the additional information stored may need to be updated (e.g., public/private keys may need to be refreshed as well as digital certificates). Data integrity YES. The federation membership should contain only the identifier of effective members. Identifiers should not be tampered or created**.
**Do you have non-repudiation requirements?**	**NO. Non-repudiation is desirable, but it is not currently a requirement**.
**Do you need to support multiple writers for the same data?**	**YES. Let us recall that the data stored are a set with all the identities of participating members. As a consequence, this set can be updated by any member that wants to leave or by a new member that is trying to join the federation**.
**Is there a TTP and is it always online?**	**NO. A TTP need to be assumed as bootstrap node to be accessed by members that want to join the federation. However, this node is not guaranteed to be always online**.
**Are writers known and trusted?**	**YES. In our context, we are considering the creation of a federation among collaboration entities. Thus, participating members must be known and trusted**.

*HCO, health-care organization; TTP, trusted third party*.

**Table 3:** Assessment of blockchain for data sharing operations.

**DATA SHARING**	
**Do you need to store data or state?**	**YES. As the name suggest, the data sharing block is going to store medical data that need to be shared among the federation**.
**Do you have immutability or data integrity requirements?**	**Immutability NO. Medical data may need to be removed from the storage to guarantee the “right to forgotten” ruled out in GDPR, or specific fields may need to be updated as stated in the “right to rectification” (contact information associated to medical data). Integrity YES. Medical data have a strong integrity requirement (also coming from GDPR). In addition, immutability is also a highly desirable property**.
**Do you have non-repudiation requirements?**	**NO. Non-repudiation is desirable, but it is not currently a requirement**.
**Do you need to support multiple writers for the same data?**	**NO. When dealing with medical data, we are considering a type of data that are produced by a data producer and then become typically read-only (e.g., blood exam reports or X-ray images are produced, and then they can just be accessed in read mode)**.
**Is there a TTP and is it always on-line?**	**YES. Without loss of generality, we can assume that the HCO that produced the medical is trusted and is always available**.
**Are writers known and trusted?**	**YES. Medical data can be produced only by HCOs, and they are assumed to be trusted and known in the federation**.

*GDPR, General Data Protection Regulation; TTP, trusted third party; HCO, health-care organization*.

Using the same process, at first, we get that blockchain is recommended to support the implementation of the data sharing mainly because it is supporting efficiently the data integrity requirement; i.e., it allows to trace data accesses and verify their authorship and integrity. We should keep in mind that medical data are mostly read-only, and in our context, they are shared between trusted parties. Thus, the main benefit we can get by adopting a blockchain-based solution is the support for data integrity verification and data auditability. However, this feature must be carefully balanced with the “right to be forgotten” requirement (i.e., a MUST requirement imposed by the GDPR regulation) and its implication on the adoption of a blockchain-based solution. In order to support the implementation of the “right to be forgotten,” we need to guarantee that data can be deleted from the blockchain when the data owner asks to do it. Currently, deleting data efficiently from a blockchain is still an open research problem, and the few existing solutions are currently based on the adoption of computationally expensive cryptographic techniques. Furthermore, when dealing with medical data, there is also the additional complexity following the huge heterogeneity of data to be considered (i.e., text, images, and images/sounds). Blockchain technologies have been originally designed to deal with transactional data, of small size and in the form of numbers or strings. Currently, it is not clear how to extend the paradigm to work with heterogeneous data. A possible solution to this issue could be to keep such heterogeneous medical data stored locally in a classical database and store in the blockchain only its hash. However, it is still not clear how much privacy lawyers consider such metadata as an expression of a personal data, and thus, the issue may remain.

## Conclusions

Information sharing in the health domain is a complex and challenging process, since there are many stakeholders involved; different and sometimes competing standards and solutions to choose from; and important security, ethics, and regulation-related constraints for any proposed solution to comply with Markakis et al. (40). HIE is a key building block for the realization of Connected Health in Europe, which “speaks to the health journey of the person, through the entire lifespan, leveraging a variety of technologies to do so” (41). Based on the information and content of this document, it is important to consider the following aspects when building a new platform for sharing medical information:

- Patient consent is of utmost importance, and infrastructure should be in place for its registration, enforcement, and withdrawal.
- Compliance with GDPR and national laws, and implementing solutions to address significant requirements, such as the “right to erasure.”
- Produce interoperable solutions by linking and interoperating with well-established standards such as document and data formats (e.g., CDA and DICOM) and metadata and value lists (e.g., SNOMED and LOINC) in order to support common understanding and integration.
- Handle the whole security spectrum: authentication and authorization of users, data privacy, auditing for “post-mortem” analysis and non-repudiation, data integrity, and machine-enforced trust among the sharing organizations.
- Enable the unique identification of patients while at the same time exposing the minimal set of personal information in order to protect their privacy.
- Performance, scalability, and availability of the whole platform should be high in order to support the health-related processes efficiently.
- Be part of the health-care ecosystem, which means allow easy integration with existing infrastructure by featuring interoperable “ports and adapters” interfaces.

Additionally, the IHE profiles should not be neglected. In the 2015/1302 Commission Decision, after consulting the European multi-stakeholder platform on information and communications technology (ICT) standardization and sectoral experts, 27 IHE profiles have been identified for referencing in public procurement, such as XDS and XDS-I, PIX, and PDQ (42).

Cloud computing is now used everywhere and provides an important set of features, such as, adaptive scalability, performance, and benefits from the business perspective. Especially for the sharing of clinical information, cloud can be very advantageous especially in cases where central repositories or central coordination are needed. But organizations should also be wary about the data protection, privacy, and access control mechanisms that should be in place (43–45), either offered by the cloud provider or built in house, in order to properly handle sensitive data and comply with regulations such as GDPR.

Blockchain is a highly interesting technology that can be put in good use in information sharing, more specifically for supporting decentralization, data integrity verification, and data auditability. These inherent features of blockchain have been praised and discussed in the context of health care as valuable tools (41, 46). Nevertheless, there are some major issues to be resolved, such as the compliance with GDPR's “right to be forgotten” requirement, which, unless the blockchain implementation is adapted, requires the deletion of data from the blockchain when the data owner asks to do it and this is not feasible, by design, in the “traditional” blockchain implementations. Furthermore, there is also the additional complexity followed by the huge heterogeneity of medical data (i.e., text and images) that do not fit exactly to the original design of blockchain. It is evident that such requirements imposed by GDPR and the application domain present challenges and introduce additional trade-offs related to the management of data, administration, and overall governance. For example, storing the health data “off-chain” (i.e., external to the blockchain network) and only metadata “on-chain” may introduce problems of availability, performance, data protection, and integrity (47). Therefore, careful considerations of the available options should be made before committing to such cutting-edge technologies.

## Data Availability Statement

The original contributions generated for the study are included in the article/supplementary material, further inquiries can be directed to the corresponding author/s.

## Author Contributions

ES, SS, SB, and CC made core contribution to the design of the study, collection of information, guidelines, established practices, and standards and made an extensive evaluation on new and promising technologies for the implementation of a secure information sharing platform for health-related data including blockchain evaluation. SM and VS coordinated the work in respect of the project PANACEA. All authors contributed to the article and approved the submitted version.

## Conflict of Interest

The authors declare that the research was conducted in the absence of any commercial or financial relationships that could be construed as a potential conflict of interest.

## Footnotes

^1^Let us note that this second set of consent could be removed if every HCO provides the TTP a copy of its data processing activity registry. However, as mentioned above, this would add the complexity of finding a good anonymization scheme that allows to preserve patients' privacy still allowing the TTP to check consent.

## References

[1] U.S. Congress. Bringing Health Care Online: The Role of Information Technologies. Washington, DC: Office of Technology Assessment (1995). p. 232.

[2] Kolodner RM Cohn SP Friedman CP. Health information technology: strategic initiatives, real progress: there is nothing magical about the strategic thinking behind health IT adoption in the United States. Health Aff. (2008) 27:w391–5. 10.1377/hlthaff.27.5.w391 18713825

[3] Spanakis EG Bonomi S Sfakianakis S Santucci G Lenti S Sorella M. Cyber-attacks and threats for healthcare – a multi-layer thread analysis. In: 2020 42nd Annual International Conference of the IEEE Engineering in Medicine & Biology Society (EMBC). Montreal, QC (2020). p. 5705–8. 10.1109/EMBC44109.2020.9176698 33019270

[4] Velsen LV Hermens H d'Hollosy WO. A maturity model for interoperability in eHealth. In: 2016 IEEE 18th International Conference on e-Health Networking, Applications and Services (Healthcom). Munich (2016). p. 1–6.

[5] Chronaki CE Chiarugi F Mavrogiannaki E Demou C Lelis P Trypakis D. An eHealth platform for instant interaction among health professionals. In: Computers in Cardiology, 2003. Thessaloniki Chalkidiki (2003). p. 101–4. 10.1109/CIC.2003.1291100

[6] Spanakis EG Lelis P Chiarugi F Chronaki C. R&D challenges in developing an ambient intelligence eHealth platform. In: EMBEC 2005, Prague, Czech Republic, November 2005. Prague (2006). vol. 11; p. 1727–983.

[7] Tsiknakis M. Spanakis M. Adoption of innovative eHealth services in prehospital emergency management: a case study. Proceedings of the 10th IEEE International Conference on Information Technology and Applications in Biomedicine. Corfu (2010). p. 1–5. 10.1109/ITAB.2010.5687752

[8] Spanakis EG Psaraki M Sakkalis V. Congestive heart failure risk assessment monitoring through internet of things and mobile personal health systems. 2018 40th Annual International Conference of the IEEE Engineering in Medicine and Biology Society (EMBC). Honolulu, HI (2018). p. 2925–8. 10.1109/EMBC.2018.8513024 30441013

[9] Spanakis M Sfakianakis S Sakkalis V Spanakis EG. PharmActa: empowering patients to avoid clinical significant drug–herb interactions. Medicines. (2019) 6:26. 10.3390/medicines6010026 PMC6473432 30781500

[10] European Commission. New European Interoperability Framework, Promoting Seamless Services and Data Flows for European Public Administrations. (2017). Available online at: https://ec.europa.eu/health/sites/health/files/ehealth/docs/ev_20151123_co03_en.pdf

[11] Katehakis DG Pangalos G Prentza A. Security improvements for better and safer cross-border ePrescription and patient summary services. Int J Reliab Qual E-Healthc. (2017) 6:18–28. 10.4018/IJRQEH.2017010102

[12] Tinholt D Carrara W Tol T Foley P Graux H Erdogan E. Study on analysis of the needs for cross-border services and assessment of the organisational, legal, technical and semantic barriers. Eur Comm-Gen Commun Netw Content Technol. (2013) 10:10003. Available online at: https://ec.europa.eu/newsroom/dae/document.cfm?doc_id=2310

[13] Peeters M. Free movement of patients: directive 2011/24 on the application of patients' rights in cross-border healthcare. Eur J Health Law. (2012) 19:29–60. 10.1163/157180912X615158 22428388

[14] Benson T. Principles of Health Interoperability HL7 and SNOMED. 2nd ed. London: Springer-Verlag (2012). 10.1007/978-1-4471-2801-4

[15] Kuchinke W Aerts J Semler SC Ohmann C. CDISC standard-based electronic archiving of clinical trials. Methods Inf Med. (2009) 48:408–13. 10.3414/ME9236 19621114

[16] Carroll R Cnossen R Schnell M Simons D. Continua: an interoperable personal healthcare ecosystem. IEEE Pervasive Comput. (2007) 6:90–4. 10.1109/MPRV.2007.72

[17] Marias K Sakkalis V Roniotis A Farmaki C Stamatakos G Dionysiou D. Clinically oriented translational cancer multilevel modeling: the contracancrum project. In: Dössel O, Schlegel WC, editors. World Congress on Medical Physics and Biomedical Engineering, September 7 - 12, 2009, Munich, Germany. IFMBE Proceedings, vol 25/4. Berlin: Springer (2009).

[18] Larobina M Murino L. Medical image file formats. J Digit Imaging. (2014) 27:200–6. 10.1007/s10278-013-9657-9 24338090 PMC3948928

[19] Cock PJA Fields CJ Goto N Heuer ML Rice PM. The Sanger FASTQ file format for sequences with quality scores, and the Solexa/Illumina FASTQ variants. Nucleic Acids Res. (2010) 38:1767–71. 10.1093/nar/gkp1137 20015970 PMC2847217

[20] Leinonen R Sugawara H Shumway M International Nucleotide Sequence Database Collaboration. The sequence read archive. Nucleic Acids Res. (2010) 39:D19–21. 10.1093/nar/gkq1019 21062823 PMC3013647

[21] Dolin RH Alschuler L Beebe C Biron PV Boyer SL Essin D. The HL7 clinical document architecture. J Am Med Inform Assoc. (2001) 8:552–69. 10.1136/jamia.2001.0080552 11687563 PMC130066

[22] Freitas F Schulz S Moraes E. Survey of current terminologies and ontologies in biology and medicine. RECIIS. (2009) 3:239–49. 10.3395/reciis.v3i1.239en

[23] Bender D Sartipi K. HL7 FHIR: an agile and RESTful approach to healthcare information exchange. In: Proceedings of the 26th IEEE International Symposium on Computer-Based Medical Systems. Porto. (2013). p. 326–31.

[24] Kondylakis H Petrakis Y Leivadaros S Iatraki G Katehakis D. Using XDS and FHIR to support mobile access to EHR information through personal health apps. In: 2019 IEEE 32nd International Symposium on Computer-Based Medical Systems (CBMS). Cordoba. (2019). p. 241–4. 10.1109/CBMS.2019.00058

[25] Eckman BA Bennett CA Kaufman JH Tenner JW. Varieties of interoperability in the transformation of the health-care information infrastructure. IBM Syst J. (2007) 46:19–41. 10.1147/sj.461.0019

[26] Nikoloudakis Y Pallis E Mastorakis G Mavromoustakis CX Skianis C Markakis EK. Vulnerability assessment as a service for fog-centric ICT ecosystems: a healthcare use case. Peer Peer Netw Appl. (2019) 12:1216–24 10.1007/s12083-019-0716-y

[27] Nofer M Gomber P Hinz O Schiereck D. Blockchain. Bus Inf Syst Eng. (2017) 59:183–7. 10.1007/s12599-017-0467-3

[28] Vergne J. Decentralized vs. distributed organization: blockchain, machine learning and the future of the digital platform. Organ Theory. (2020) 1. 10.1177/2631787720977052

[29] Macdonald M Liu-Thorrold L Julien R. The blockchain: a comparison of platforms and their uses beyond bitcoin. Work Pap. (2017) 3:1–8. 10.13140/RG.2.2.23274.52164

[30] Kuo T-T Rojas HZ Ohno-Machado L. Comparison of blockchain platforms: a systematic review and healthcare examples. J Am Med Inform Assoc. (2019) 26:462–78. 10.1093/jamia/ocy185 30907419 PMC7787359

[31] Chowdhury MJM Ferdous MS Biswas K Chowdhury N Kayes ASM Alazab M. A comparative analysis of distributed ledger technology platforms. IEEE Access. (2019) 7:167930–43. 10.1109/ACCESS.2019.2953729

[32] Triantafyllou A Jimenez JAP Torres ADR Lagkas T Rantos K Sarigiannidis P. The challenges of privacy and access control as key perspectives for the future electric smart grid. IEEE Open J Commun Soc. (2020) 1:1934–60. 10.1109/OJCOMS.2020.3037517

[33] Radoglou Grammatikis P Sarigiannidis P Efstathopoulos G Panaousis E. ARIES: a novel multivariate intrusion detection system for smart grid. Sensors. (2020) 20:5305. 10.3390/s20185305 32948064 PMC7570496

[34] Chockler GV Keidar I Vitenberg R. Group communication specifications: a comprehensive study. ACM Comput Surv. (2001) 33:427–69. 10.1145/503112.503113

[35] Aguilera MK Keidar I Malkhi D Shraer A. Dynamic atomic storage without consensus. J ACM. (2011) 58:1–32. 10.1145/1944345.1944348

[36] Coiera E Clarke R. e-Consent: the design and implementation of consumer consent mechanisms in an electronic environment. J Am Med Inform Assoc. (2004) 11:129–40. 10.1197/jamia.M1480 14662803 PMC353020

[37] Bergmann J Bott OJ Hoffmann I Pretschner DP. An eConsent-based system architecture supporting cooperation in integrated healthcare networks. Stud Health Technol Inform. (2005) 116:961–6. 16160382

[38] Bergmann J Bott OJ Pretschner DP Haux R. An e-consent-based shared EHR system architecture for integrated healthcare networks. Int J Med Inf. (2007) 76:130–6. 10.1016/j.ijmedinf.2006.07.013 16971171

[39] Staderini M Schiavone E Bondavalli A. A requirements-driven methodology for the proper selection and configuration of blockchains. In: 2018 IEEE 37th Symposium on Reliable Distributed Systems (SRDS). Salvador: IEEE (2018). p. 201–6. 10.1109/SRDS.2018.00031

[40] Markakis E Nikoloudakis Y Pallis E Manso M. Security assessment as a service cross-layered system for the adoption of digital, personalised trusted healthcare. In: 2019 IEEE 5th World Forum on Internet of Things (WF-IoT). Limerick: IEEE (2019). p. 91–4. 10.1109/WF-IoT.2019.8767249

[41] Angraal S Krumholz HM Schulz WL. Blockchain technology applications in health care. Circ Cardiovasc Qual Outcomes. (2017) 10:e003800. 10.1161/CIRCOUTCOMES.117.003800 28912202

[42] European Commission. Commission Decision (EU) 2015/1302 on the identification of ‘Integrating the Healthcare Enterprise' profiles for referencing in public procurement. (2015). Available online at: https://data.europa.eu/eli/dec/2015/1302/oj

[43] Spanakis EG Spanakis M Karantanas A Marias K. Secure access to patient's health records using SpeechXRays a mutli-channel biometrics platform for user authentication. In: 2016 38th Annual International Conference of the IEEE Engineering in Medicine and Biology Society (EMBC). Orlando, FL (2016). p. 2541–4. 10.1109/EMBC.2016.7591248 28268840

[44] Radoglou Grammatikis PI Sarigiannidis PG Moscholios ID. Securing the internet of things: challenges, threats and solutions. Internet Things. (2019) 5:41–70. 10.1016/j.iot.2018.11.003

[45] Pasquale F Ragone T. Protecting Health Privacy in an Era of Big Data Processing and Cloud Computing. Faculty Scholarship (2014). Available online at: https://digitalcommons.law.umaryland.edu/fac_pubs/1542

[46] Linn LA Koo MB. Blockchain for health data and its potential use in health it and health care related research. In: ONC/NIST Use of Blockchain for Healthcare and Research Workshop. Gaithersburg, MD: ONC/NIST (2016). p. 1–10.

[47] Miyachi K Mackey TK. hOCBS: a privacy-preserving blockchain framework for healthcare data leveraging an on-chain and off-chain system design. Inf Process Manage. (2021) 58:102535. 10.1016/j.ipm.2021.102535

